# Pontic Site Development in Esthetic Implant Therapy: The Root Submergence Technique

**DOI:** 10.7759/cureus.79330

**Published:** 2025-02-19

**Authors:** Mohammad Ali Alshahrani, Issa A Abu Amara, Sultan J Alqahtani

**Affiliations:** 1 Prosthodontics, Prince Abdulrahman Advanced Dental Institute, Riyadh, SAU; 2 Prosthodontics, Armed Forces Hospital Southern Region, Khamis Mushait, SAU

**Keywords:** aesthetic, gingival recession, implant, pontic site, root submergence

## Abstract

Improving the aesthetic outcome of dental implants, especially in the anterior maxillary region, presents a considerable clinical challenge. This case report investigates the utility of a root submergence technique (RST) to address aesthetic concerns associated with fixed dental prostheses in this crucial area. Two female patients, aged 45 and 50 years, sought treatment for smile aesthetics and concerns related to gingival recession and spacing. The RST was employed to address these issues and enhance aesthetic outcomes. Our experience suggests that the cautious application of the RST holds promise for improving the aesthetic appearance of pontic sites in the anterior maxillary region. However, further research, including longitudinal follow-up studies, is essential to ascertain the long-term effectiveness and stability of this approach.

## Introduction

Despite the high long-term success rate of implant prostheses, implant restorations in the anterior dentition may present aesthetic challenges [1]. When a tooth is removed, the natural process of alveolar ridge resorption begins and persists over the patient's lifetime. While tooth extraction typically heals without complications, research indicates that the loss of a tooth leads to resorption of the alveolar ridge, particularly within the first year post-extraction [2]. According to Carlsson and Persson, immediate dentures can result in a 25% reduction in alveolar bone width and a 4 mm decrease in vertical height during the first year after extraction [3]. The loss of supporting bone leads to the migration of soft tissues towards the apex, resulting in the formation of unattractive black triangles. This not only concerns aesthetics but also affects speech and can lead to food getting stuck between teeth [4]. Currently, several techniques, including the scalloped implant and platform switching, have been developed in an attempt to control alveolar bone resorption and preserve crestal bone height around adjacent implants. These approaches have not consistently provided satisfactory results in overcoming aesthetic challenges [5,6].

The root submergence technique (RST) conserves the periodontal tissues surrounding a tooth root, eliminating the need for extraction [7]. By submerging the root, the periodontal attachment complex remains intact, preventing the loss of alveolar bone on both the outer and inner walls, as well as in the spaces between teeth [8]. Consequently, this approach preserves the size of the alveolar ridge and the neighboring tissues [9].

In 1992, the initial instance of guided bone regeneration (GBR) near dental implants was documented, marking the beginning of the GBR era, as commercial biomaterials gained traction [10]. Over time, researchers have observed remarkable outcomes in long-term follow-ups of 20 to 30 years [11]. Three decades later, pivotal reports advanced the application of root-mediated ridge preservation in implant dentistry. One report involved submerging an incisor root in a child to promote replacement resorption for future implant placement [12], while another report detailed the submerging of roots beneath implant-supported fixed partial dentures (FPDs), as described by Salama et al. [8].

Clinical and histological investigations have revealed that non-infected, vital, or endodontically treated roots, positioned at or below the level of the bone crest and fully submerged within the alveolar socket, effectively maintain the integrity of the residual ridge [13]. This approach has recently been extended to the rehabilitation with fixed dental prostheses. In cases where a pontic is required, a root is maintained at the crestal bone level to preserve the ridge morphology, with no anticipated root exposure due to the absence of direct pressure [7]. Additionally, the RST has found application in implant-supported prostheses in the anterior region of the upper jaw [9].

This case report delves into the impact of employing an RST in the preservation of periodontal tissue specifically at the pontic site within the aesthetic zone of the mouth. It details the methodology and outcomes of utilizing this technique, shedding light on its efficacy in maintaining the health and integrity of the surrounding periodontium in a region crucial for aesthetics.

## Case presentation

Case 1

Patient Presentation

**Figure 1:**
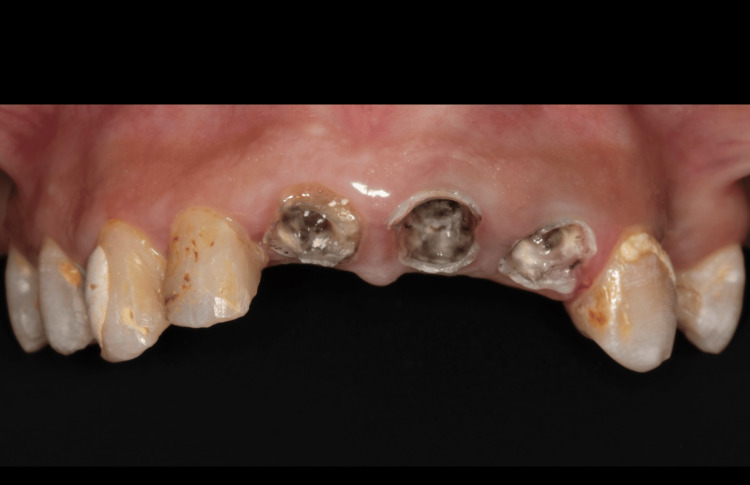
Clinical preoperative view.

A 45-year-old Saudi female patient presented with concerns regarding her smile aesthetics at the Dental Department, Prince Abdulrahman Advanced Dental Institute, Riyadh, Saudi Arabia, in September 2021. The patient was medically fit and had a past dental history of endodontic treatment of front teeth. The clinical examination and diagnostic radiographic evaluation revealed that teeth #11, #21, and #22 had severe caries and a history of endodontic treatment (Figure 1).

Treatment Plan Formulation

**Figure 2:**
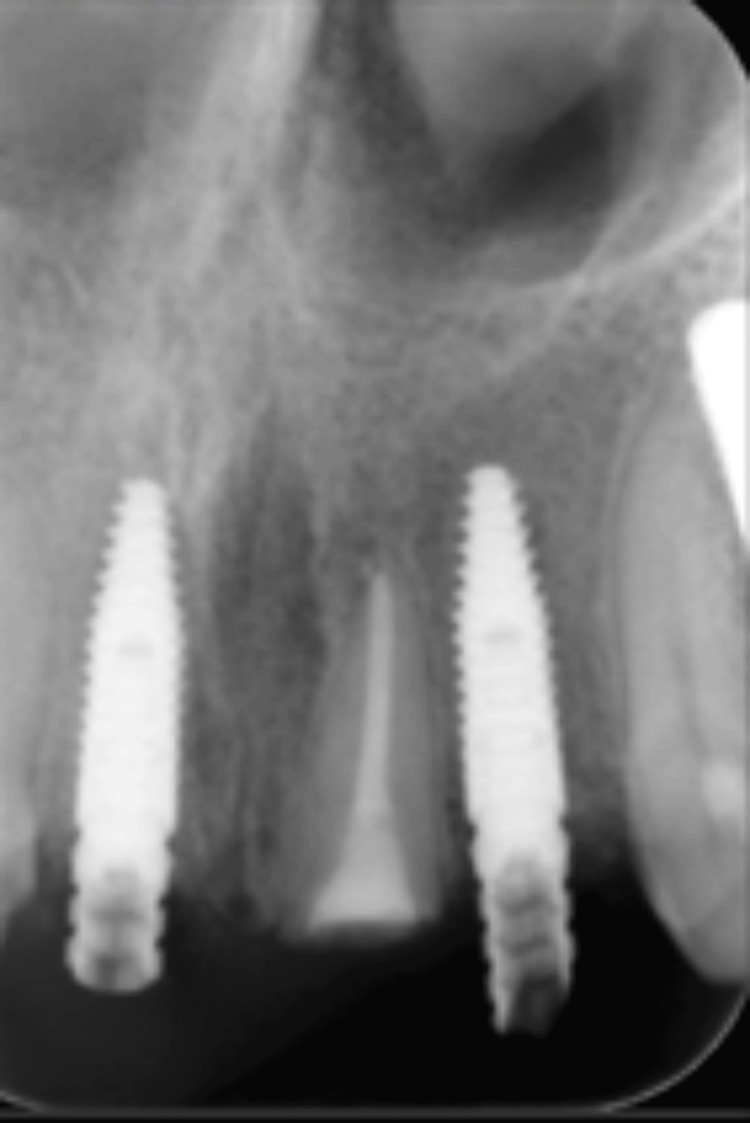
Radiograph view of the submerged root.

**Figure 3:**
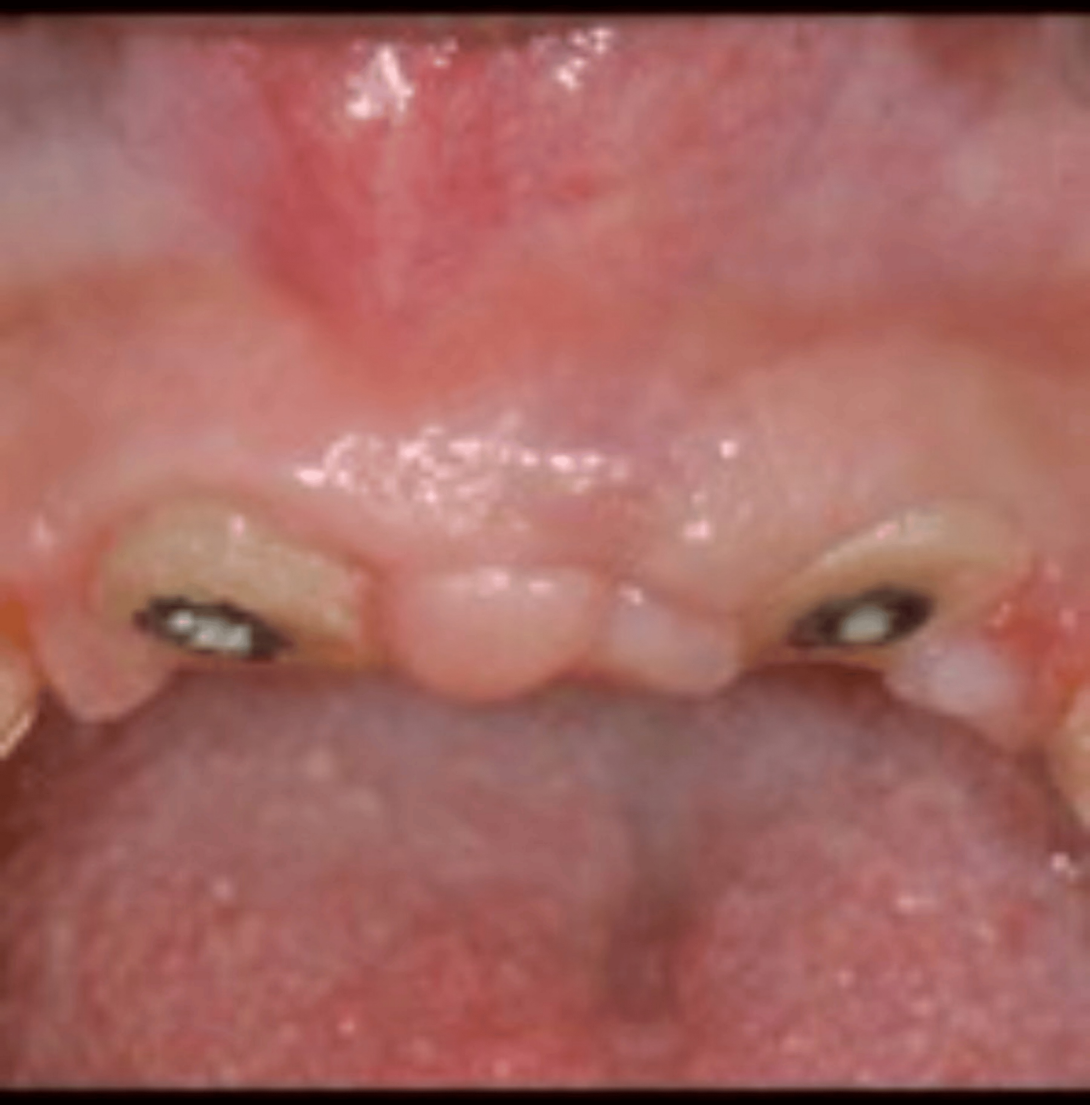
Clinical view of the customized healing abutment after the extraction of teeth #11 and #22.

Due to the non-restorable nature of the carious teeth, a comprehensive treatment plan was devised to restore function and aesthetics while preserving the underlying bony architecture. The plan included extracting teeth #11 and #22, followed by ridge preservation using a cancellous particulate allograft with a resorbable collagen membrane, employing the ice cream cone technique. Additionally, endodontic therapy was recommended for tooth #21, followed by a root submergence procedure to position the root 2 mm below the crestal bone level (Figures 2, 3).

Implementation and Follow-Up

**Figure 4:**
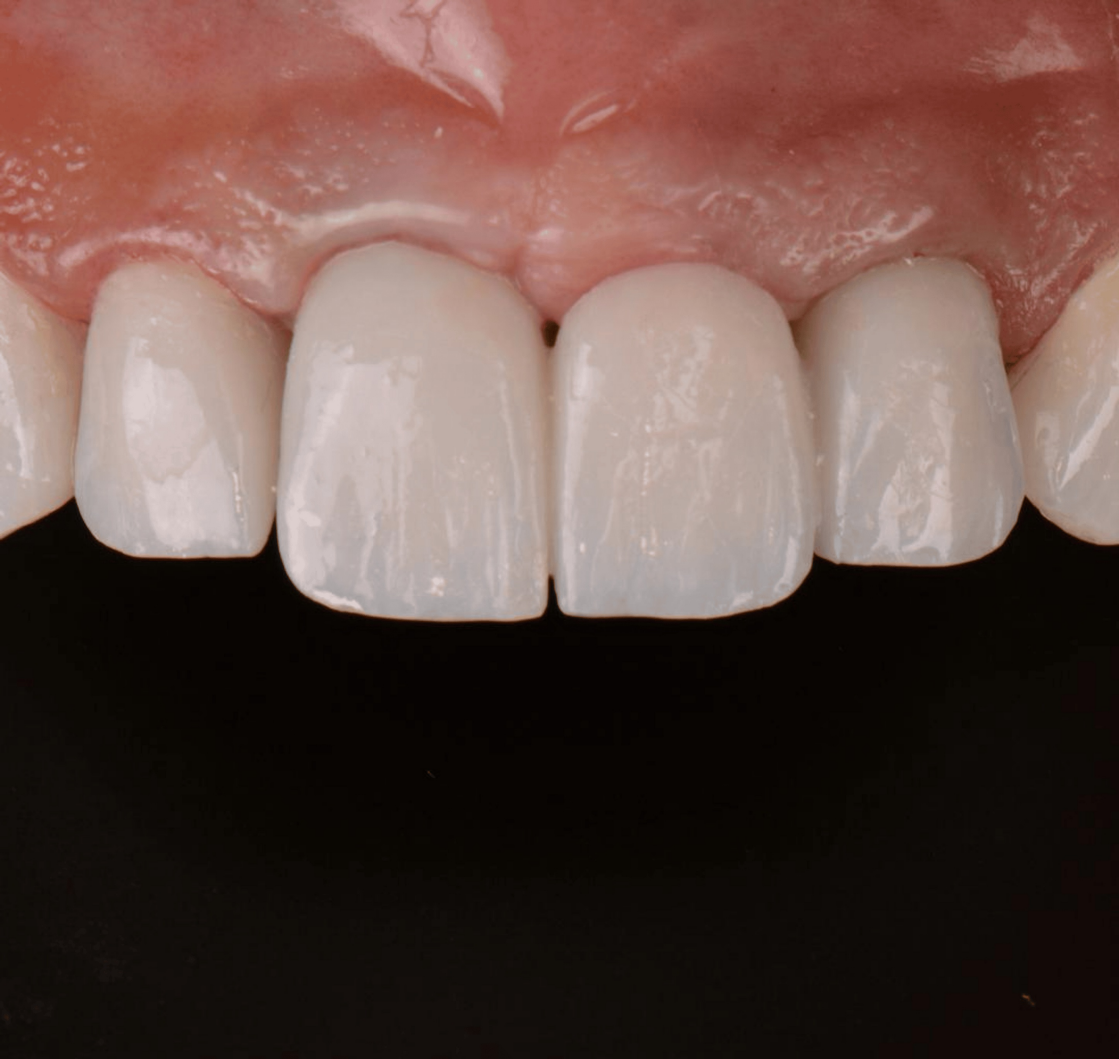
Clinical view of the implant-supported, screw-retained provisional prosthesis.

**Figure 5:**
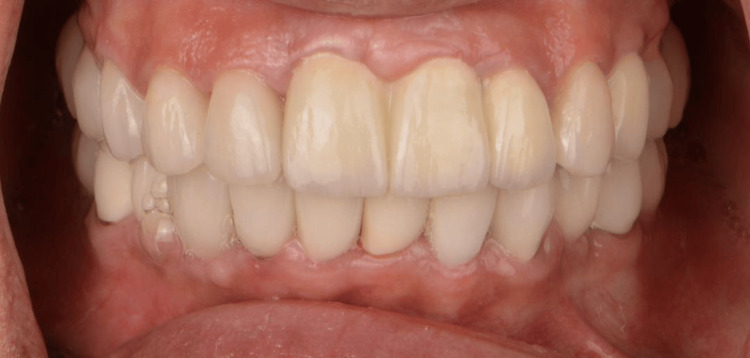
Clinical view of the final prosthesis.

After obtaining written consent from the patient, teeth #11 and #22 were extracted, and tooth #21 underwent decoronation. After approximately six months of healing following ridge preservation in the #11 and #22 areas, implant placement was performed using two 3.3 × 12 mm Straumann bone-level tapered Roxolid implants. Customized healing abutments were utilized to facilitate optimal healing and soft tissue contouring (Figures 4, 5).

Case 2

Patient Profile

A 50-year-old Saudi female patient who underwent full-mouth rehabilitation, including implants six years ago, presented to the Dental Department at Prince Abdulrahman Advanced Dental Institute in Riyadh, Saudi Arabia, in October 2023, with concerns about gingival recession and spacing in the upper anterior segment.

Clinical Assessment

**Figure 6:**
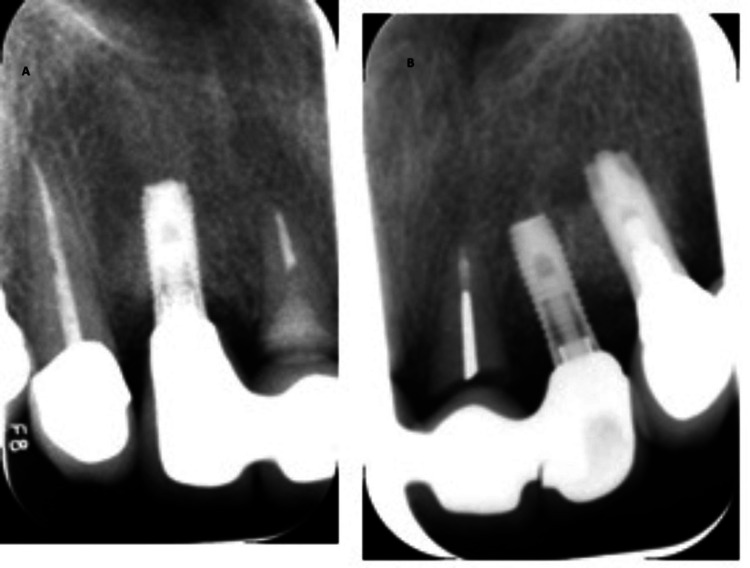
Preoperative radiographic view of (A) tooth #11 and (B) tooth #21.

Upon clinical and radiographic examination, it was observed that the recession of the interdental papillae had led to the formation of black triangles between the four upper incisors. Additionally, carious lesions extending to the root were identified in both central incisors (#11 and #21) (Figure 6).

Treatment Strategy

**Figure 7:**
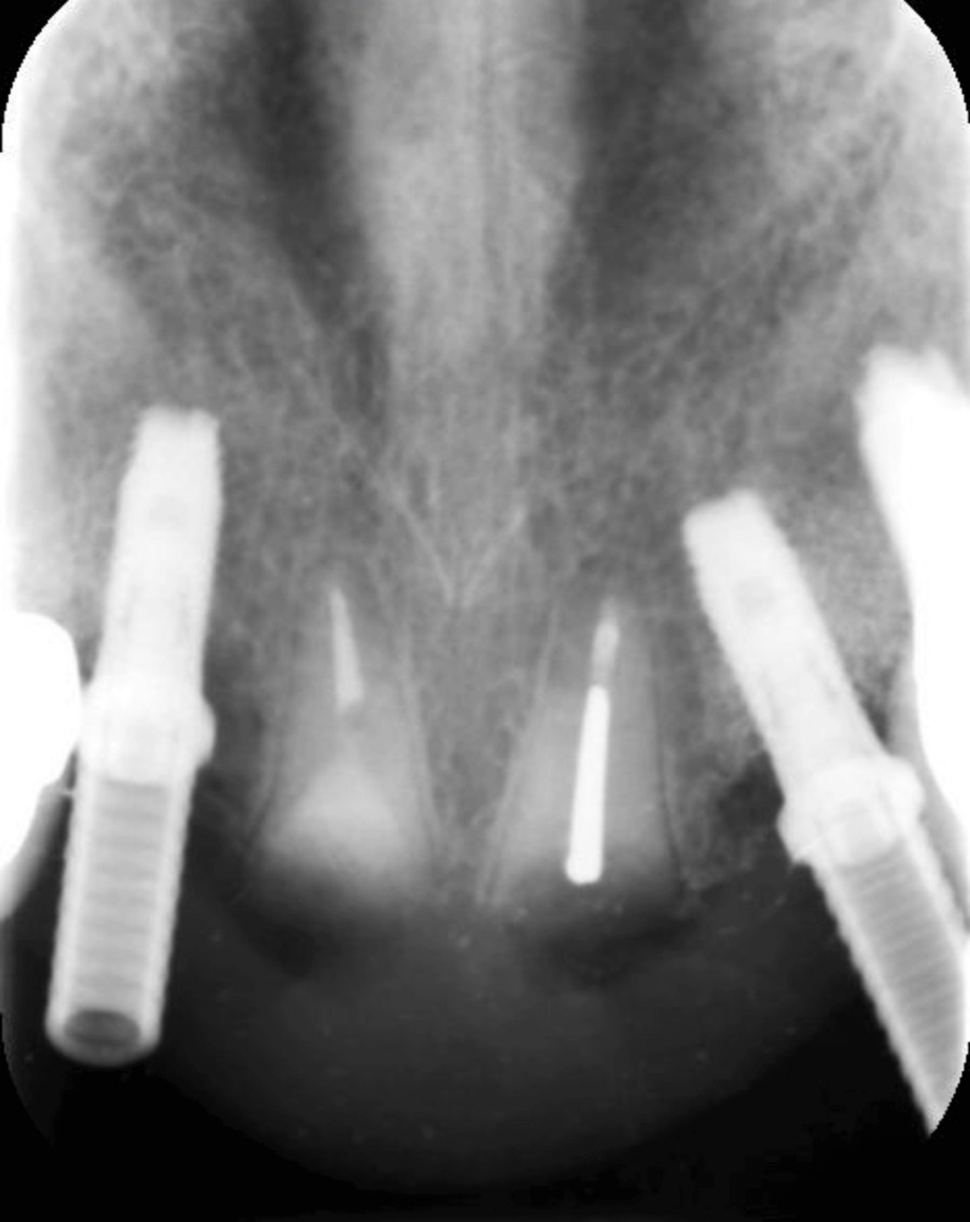
Periapical (PA) radiographic view of the submerged roots of teeth #11 and #21.

After obtaining written consent to address the patient's concerns and preserve the ridge contours while enhancing aesthetics, a treatment plan was devised. The plan involved decoronation of both central incisors, caries excavation, and composite restoration. Subsequently, the roots were submerged 2 mm below the crest of the bone (Figure 7).

Implementation and Restoration

**Figure 8:**
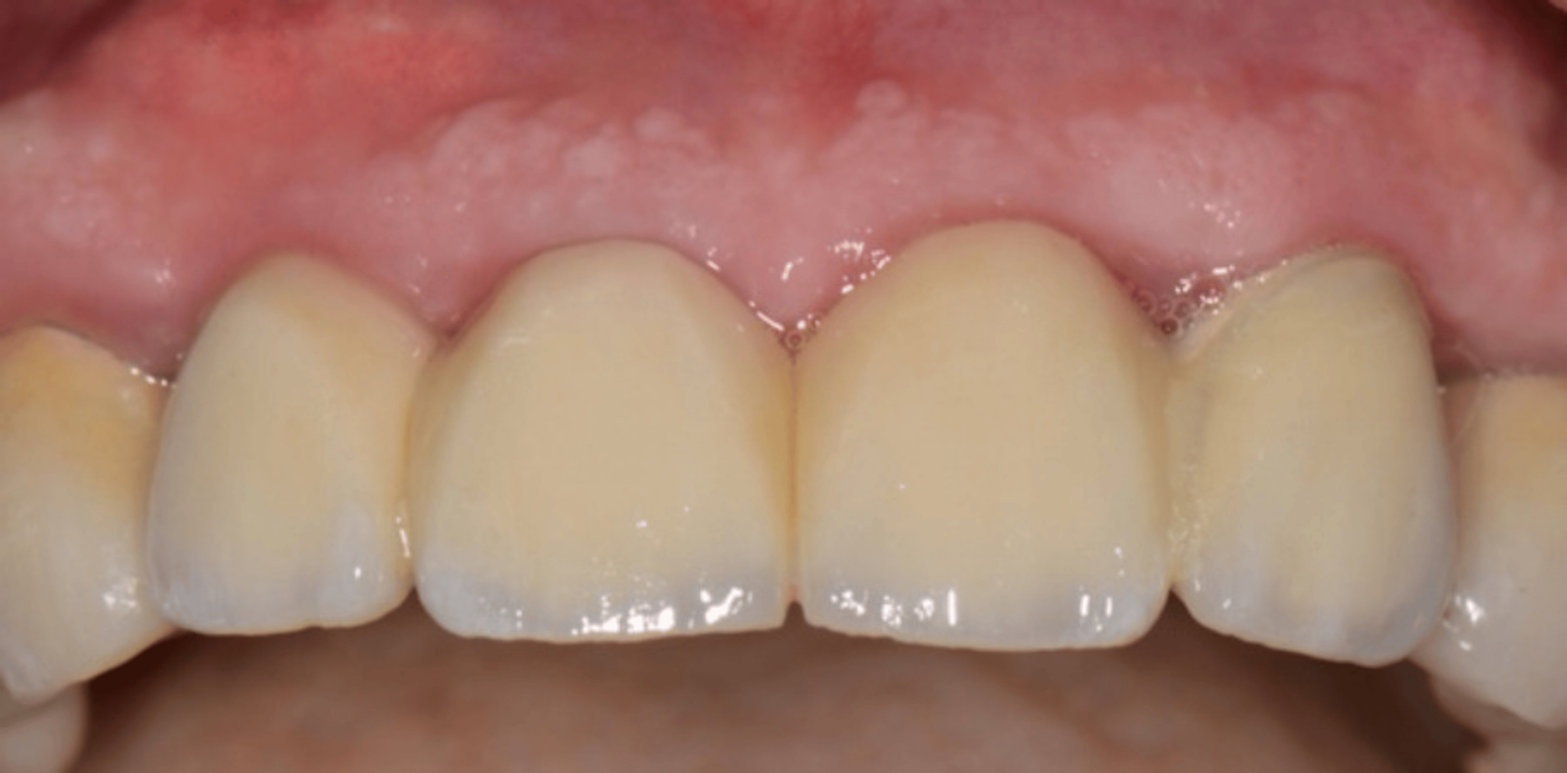
Clinical view of the final prosthesis.

**Figure 9:**
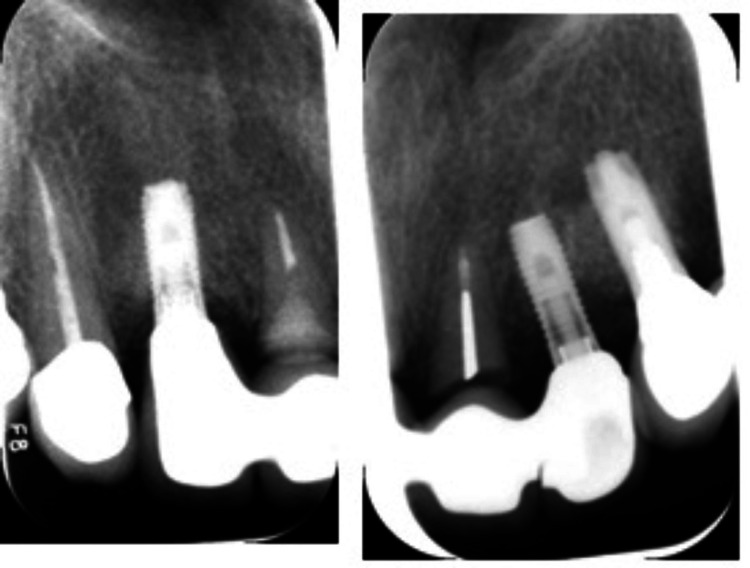
Periapical (PA) radiographic view of the final prosthesis.

Following the decoronation and root submergence procedures, teeth #11 and 21 were replaced with an implant-supported fixed dental prosthesis (FDP). The FDP was supported by implants in the #12 and #22 areas, providing stability and functionality for the restored anterior segment (Figures 8, 9).

## Discussion

Residual ridge atrophy after the extraction of a non-salvageable tooth poses significant challenges to achieving satisfactory aesthetic results, particularly in the maxillary anterior region. Following tooth extraction, the process of residual ridge remodeling initiates, accompanied by an inflammatory response cascade [14]. Despite ongoing bone deposition within the extraction socket over several months, it typically fails to reach the coronal bone level of adjacent teeth, as evidenced by research conducted by Schropp et al. [15].

If a hopeless tooth lacks periapical pathology, its remaining root can be submerged to maintain the health of the surrounding periodontal tissues [16]. Despite the availability of various surgical approaches for augmenting soft and hard tissues to address aesthetic concerns with implant prostheses, it's important to note that some aesthetic issues associated with implants may not be entirely correctable [17].

The RST initially emerged as a means to conserve the periodontal attachment complex, aiming to enhance the retention and stability of removable prostheses. Numerous studies have documented the successful preservation of periodontal tissues when utilizing this technique, whether the roots are vital or have undergone endodontic treatment, and when they are covered by bone or soft tissue [16]. Additionally, submerged roots positioned between dental implants have been shown to uphold gingival architecture and prevent interproximal bone resorption [9].

In some complex defects, the use of the RST following orthodontic extrusion of the remaining root has been extremely successful in establishing the ideal pontic form. The RST allows for an improved esthetic result with long-term predictability when restoring multiple adjacent teeth in the esthetic zone [8].

von Wowern and Winther conducted a four-year follow-up study on 20 cases involving crown-resected, endodontically treated roots. They noted gingival tissue perforation in 11 cases, classified as failures. Preservation of the alveolar ridge was often compromised when roots were submerged and dentures were placed. Conversely, there are no documented instances of gingival perforation or exposure related to pontic sites where occlusal forces do not directly impact the gingiva [18]. Harper successfully followed up on a case involving submerging an endodontically treated root in the anterior maxilla, maintaining the site, termed as *submerged root/pontic*, over six years [7].

The RST emerges as an effective method for preventing bone resorption at the pontic site. This approach not only maintains the alveolar bone but also facilitates the creation of an ideal interdental papilla that harmonizes in color, form, and contour with adjacent tissues. In a study by Rodd et al. in 2002, the retention of permanent anterior roots in young individuals was supported, given the high clinical success rate exceeding 90% over two years [19]. Additionally, Salama et al. proposed a strategy aimed at establishing a more predictable protocol for esthetic implant treatment for multiple-tooth defects utilizing the RST approach [8].

Strengths 

The use of the RST represents a novel approach to aesthetic implant therapy, showcasing advancements in clinical practices. The case report addresses a common challenge in dental aesthetics, providing insights that can be beneficial for practitioners dealing with similar cases. Reporting on patient satisfaction and aesthetic results highlights the importance of patient perspectives in clinical decision-making. Long-term follow-up data provide valuable insights into the durability and success of the technique over time. The report adds to the existing literature on aesthetic implant therapy, potentially guiding future research and clinical practices.

Limitations 

As a case report, findings may not be generalizable to larger populations or diverse clinical settings. The absence of a control group limits the ability to compare outcomes and assess the effectiveness of the RST against alternative methods. Evaluation of aesthetic outcomes can be subjective, influenced by personal preferences and biases of both the clinician and the patient.

## Conclusions

In conclusion, the RST presents itself as a promising avenue for preserving the pontic site within the esthetic zone. However, it is imperative to proceed with caution and meticulous attention to detail when implementing this technique in clinical practice. While existing evidence suggests favorable short-term outcomes, further longitudinal follow-up studies are essential to comprehensively assess the long-term efficacy and sustainability of this approach. By conducting more extensive and extended studies, clinicians and researchers can gain deeper insights into the durability, stability, and aesthetic benefits conferred by the RST, thereby enhancing its applicability and refining treatment protocols for optimal patient outcomes.
